# Posterior aortoventricular enlargement of root and outflow tract: Early and mid-term results

**DOI:** 10.1016/j.xjtc.2025.10.001

**Published:** 2025-10-13

**Authors:** Karen B. Abeln, Idriss Souko, Jochen Pfeifer, Christian Giebels, Hans-Joachim Schäfers

**Affiliations:** a Department of Cardiovascular Surgery, Saarland University Medical Center and Saarland University, Homburg, Germany; b Department of Pediatric Cardiology, Saarland University Medical Center, Homburg, Germany; c Department of Thoracic and Cardiovascular Surgery, Westpfalz Hospital, Kaiserslautern, Germany; d Professor Emeritus, Department of Cardiovascular Surgery, Saarland University, Saarbrücken, Germany

**Keywords:** pulmonary autograft, aortoventricular enlargement, aortic valve replacement

## Abstract

**Objective:**

Congenital hypoplasia of the left ventricular outflow tract (LVOT) and aortic annulus is a challenging problem. We have developed a novel posterior enlargement technique that avoids a septal and right ventricular incision while effectively enlarging the LVOT and aortic annulus. Here we report mid-term results with the use of this technique.

**Methods:**

Between 2011 and 2023, 5 patients (3 males [60%]; mean age, 28 ± 23 years) underwent posterior aortoventricular enlargement. The aortic valve and root were replaced by an autograft (n = 4) or a stentless porcine prosthesis (n = 1).

**Results:**

There were cases of no early death, myocardial infarction, neurologic complications, or permanent pacemaker implantation. One patient died 3 years postoperatively in a traffic accident, and 1 patient required pulmonary conduit reoperation for infective endocarditis 6.5 years postoperatively. Freedom from cardiac death and aortic valve or root reoperation was 100% at 10 years. Two patients had AR 1 at discharge, and 3 patients had AR 1 at last follow-up, between 12.1 and 13.4 years postoperatively, including the 2 with AR 1 at discharge. Median LVOT diameter was 12 mm preoperatively (interquartile range [IQR], 9.5-12.6 mm), 20 mm (IQR, 20-21 mm) at discharge, and 20 mm (IQR, 20-21 mm) at last follow-up. Median annular diameter was 17 mm (IQR, 15-18 mm) preoperatively, 21 mm (IQR, 20-22 mm) at discharge, and 25 mm (IQR, 24-26 mm) at last follow-up. Median LVOT gradient was reduced from 50 mm Hg (IQR 41-57 mm Hg) preoperatively to 9 mm Hg (IQR, 7-16 mm Hg) at discharge. All patients remained in sinus rhythm with normal PR intervals and QRS complexes.

**Conclusions:**

Posterior aortoventricular enlargement combined with root replacement yields effective relief of LVOT and annular hypoplasia. It appears to be a durable option for younger and older patients alike.

**Figure:**
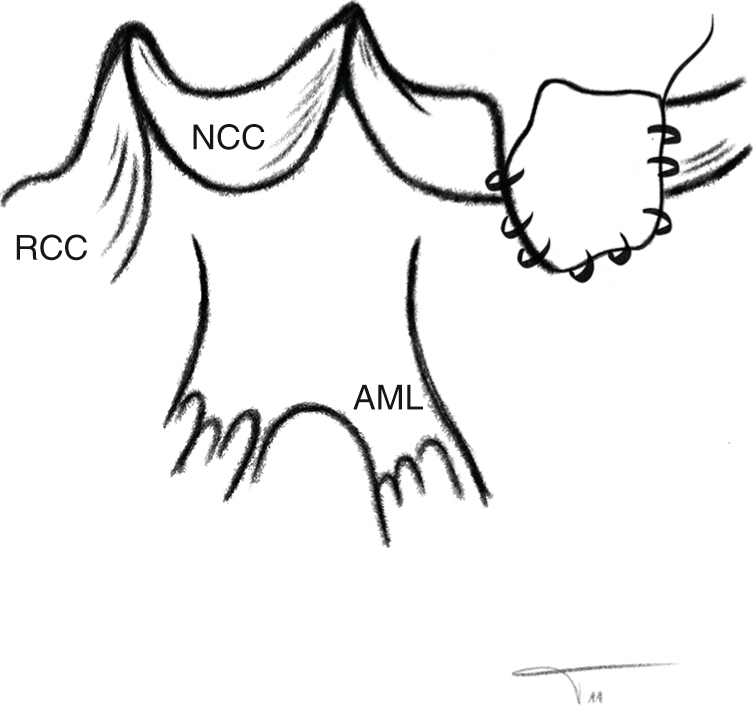
Drawing of the cusps and mitral continuity, with a patch inserted in the high-anterolateral wall.

**Central Message**

Posterior enlargement effectively opens the aortic annulus and left ventricular outflow tract and avoids a septal and right ventricular incision combined with root replacement.

**Perspective**

Hypoplasia of the left ventricular outflow tract and aortic annulus is a challenging problem. Posterior enlargement is a new and durable option for this pathology. Combined with root replacement, it is effective without interfering with left ventricular function or the conduction system.

Aortic valve replacement has been the standard treatment of aortic valve stenosis and generally leads to good hemodynamic results. In some patients, however, the annular diameter may limit the size of the prosthesis that can be implanted. This can lead to patient–prosthesis mismatch with elevated gradients at rest and even more so during exercise.^1^ As a consequence, left ventricular workload remains elevated, left ventricular mass regression is limited, and long-term survival may be reduced.^2^^,^^3^ Root replacement often allows for implantation of a larger prosthesis, and the best hemodynamics have been obtained with a pulmonary autograft^4^ or stentless biological prosthesis if implanted as a root replacement.^5^, ^6^, ^7^ Alternatively, different techniques of aortic annular enlargement have been proposed to allow implantation of larger prostheses.^8^, ^9^, ^10^, ^11^, ^12^, ^13^ Recently, an aggressive annular enlargement technique has been proposed,^12^ allowing for implantation of even larger valve substitutes. The size of the left ventricular outflow tract LVOT will still limit outflow, however.

Occasionally, annular hypoplasia may occur in conjunction with hypoplasia of the LVOT. Such an LVOT obstruction poses a particular problem for the surgeon, because valve replacement with annular enlargement will not address the stenotic component of the LVOT, and other procedures must be performed to enlarge the LVOT. An accepted approach is the Ross-Konno procedure, in which the aortic annulus and LVOT are incised and enlarged in their septal parts.^10^^,^^13^ Insertion of the patch narrows the right ventricular outflow tract (RVOT), which also must be enlarged with a patch. The autograft is implanted into the enlarged annulus.^14^ The septal intervention of the procedure may lead to interference with the conduction system and adversely affect left ventricular function.^15^ There is a relevant risk for atrioventricular block with a need for pacemaker implantation in up to 30% of cases.^10^^,^^16^^,^^17^ In addition, while LVOT obstruction is effectively alleviated acutely, recurrent LVOT obstruction may occur over time.^18^^,^^19^ Survival is acceptable but not good (82.9% at 5 years and 65.5% at 15 years^18^).

Mavroudis and colleagues^10^ described a modified posterior incision to achieve limited enlargement of the aortic annulus before implanting the pulmonary autograft. This avoids interference with the conduction system but in its original form has no effect on the size of the LVOT.

Stimulated by the limited posterior annular enlargement, which avoids interference with the mitral apparatus,^10^ we have developed a more aggressive form of combined enlargement of annulus and LVOT by carrying the incision further and performing posterior patch enlargement. This procedure spares the septum and thus avoids negative consequences of the Konno procedure. It not only increases annular size, but also enlarges the LVOT and thus addresses LVOT hypoplasia. In this report, we analyze the early and long-term results of this technique.

## Methods

### Patients

We conducted this retrospective analysis of patients who underwent dilatation of the posterior annulus and LVOT as part of a root replacement procedure at Saarland University Medical Center between June 2011 and July 2023.

### Ethics Statement

The investigation was approved by the Saarland Regional Ethics Committee (CEP 203/19; approved May 15, 2018), and individual patient consent was waived for the analysis and publication of anonymized data.

### Inclusion Criteria

Patients who required surgical treatment of aortic stenosis in the presence of a hypoplastic LVOT were included. The LVOT and annulus were considered hypoplastic in adults when measuring <15 mm (indexed ≤8 mm/m^2^) and <19 mm (indexed ≤9 mm/m^2^), respectively. They were considered hypoplastic in children with a *z*-score ≤−2. To exclude the size-limiting effect of valvular aortic stenosis on the LVOT, the size was confirmed after excision of the valve and mobilization of the coronary ostia.

### Surgical Technique

**Figure 1:**
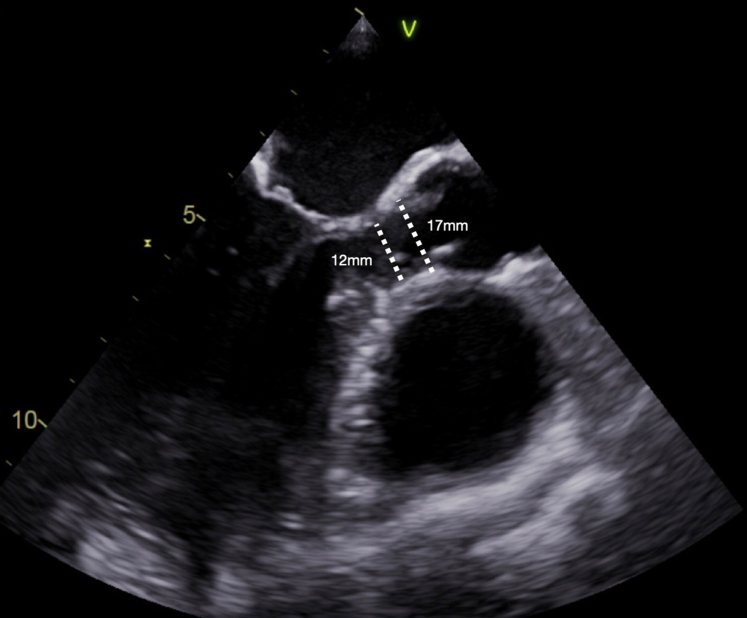
Transesophageal echocardiography, preoperative long-axis view showing the aortic valve and left ventricular outflow tract (in systole) and its diameters.

Preoperatively, the size of the LVOT and the annular diameter were determined by transesophageal echocardiography (Figure 1, Video 1). The chest was opened by median sternotomy, and the patient was connected to cardiopulmonary bypass using aortic and right atrial cannulation. The aorta was transected approximately 10 mm above the sinotubular junction. Blood cardioplegia was administered directly into the coronary ostia.

**Figure 2:**
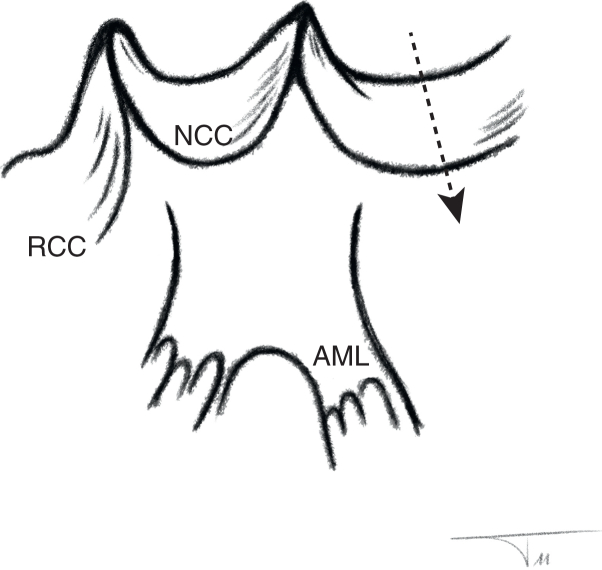
Drawing of the anatomy of the cusps and mitral continuity showing the incision that will be made in the high lateral wall (*arrow*) deep into the left ventricular outflow tract. *AML*, Anterior mitral leaflet; *NCC*, non-coronary cusp; *RCC*, right coronary cusp.

In all cases, the decision for root replacement was made to achieve ideal hemodynamics. The coronary ostia were mobilized, and the noncoronary sinus was incised (Figure 2, Video 1). After excision of the aortic valve, subvalvular fibrotic tissue or myocardium was removed from the septum if present. Annular and LVOT size were then measured using a graded Hegar dilator, and the operation was limited to root replacement only in patients with an LVOT size of ≥19 mm. In patients with an LVOT diameter <19 mm, the operation was continued with LVOT enlargement as planned.

**Figure 3:**
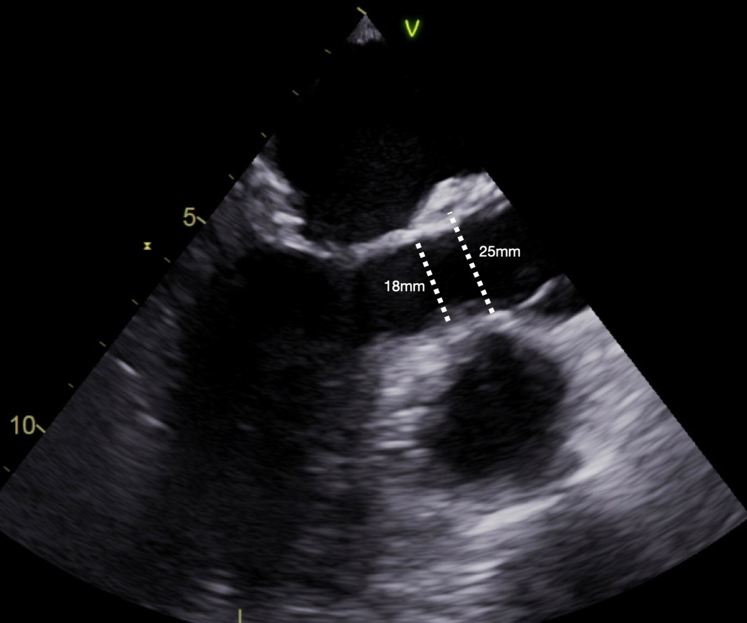
Transesophageal echocardiography, postoperative long-axis view showing the aortic valve and left ventricular outflow tract (in systole) and its enlarged diameters.

As described previously,^20^ LVOT enlargement was done via a posterior incision through the annulus and into the high lateral left ventricular wall inferior to the original location of the left coronary artery, that is, the myocardium between mitral valve tissue and septum (Figure 2, Video 1). The incision was carried into the muscular ventricular wall for a length of approximately 2 cm (Figure 2, Video 1), immediately opening the space between the mitral valve and septum. A triangular pericardial patch was then trimmed and inserted into the ventricular incision using a running suture (polypropylene 4-0; Video 1). Using this technique, the LVOT diameter was enlarged by 6 mm to 10 mm (Figure 3).

The root replacement technique with a stentless biological prosthesis was performed in standard fashion; details of the full root technique for pulmonary autograft replacement have been published previously.^21^ The proximal suture line was placed intraannularly connecting the valve substitute to the LVOT and posteriorly to the patch. Continuous sutures were used in all cases. The pulmonary autograft was supported using the native aortic wall remnants.^21^

### Statistical Analysis

Non-normally distributed variables are presented as median (interquartile range [IQR]); categorical variables, as frequency (%). Time-dependent data were assessed using the Kaplan-Meier method and the log-rank test. Survival and freedom from reoperation were calculated at 5, 10, and 15 years. Statistical analyses were performed using SPSS 28.0 (IBM).

### Follow-up

All patients were seen regularly by their referring cardiologists or in our clinic. Echocardiograms from our institution and referring cardiologists were reviewed. All patients were followed prospectively both clinically and echocardiographically (at discharge, 3 months, 1 year, and yearly thereafter). Mean gradients were measured using continuous-wave Doppler. Regurgitation was determined using color Doppler according to European guidelines. The median duration of follow-up was 12.5 years (IQR, 5.8-12.8 years). Follow-up was 100% complete.

## Results

### Patients

The study cohort comprised 5 patients (3 males), ranging in age from 7 years to 62 years (median, 21 years; IQR, 10-42 years) (Table 1). Three patients had isolated AS, and the other 2 patients had combined aortic valve disease. Valve morphology was unicuspid in 2 patients, bicuspid in 1 patient, and tricuspid in 2 patients. The median preoperative LVOT gradient was 51 mm Hg (IQR, 46-54 mm Hg).

**Table 1:** Patient characteristics

Patient	Sex	Age, y	Prior surgery	Indication	Annulus, mm	Annulus indexed, mm/m^2^	Sinus, mm	STJ, mm	Valve morphology	LVOT, mm	LVOT indexed, mm/m^2^	Mean LVOT gradient	Annulus *z*-score
1	Male	10	Yes	AS	17	-	21	20	Bicuspid	12	-	35	−2
2	Male	42	Yes	AS	19	8.7	28	22	Tricuspid	10	5.1	51	-
3	Female	21	Yes	AS	15	9	20	20	Unicuspid	15	8	32	-
4	Male	7	Yes	AR/AS	15	-	20	19	Unicuspid	8	-	64	−2.1
5	Female	62	Yes	AR/AS	17	8.8	25	23	Tricuspid	12	6.6	50	-

*STJ*, Sinotubular junction; *LVOT*, left ventricular outflow tract; *AS*, aortic stenosis; *AR*, aortic regurgitation.

All 5 patients underwent posterior patch enlargement of aortic annulus and LVOT; 4 patients had a pulmonary autograft inserted in aortic position, and 1 patient received a stentless biological substitute (Medtronic Freestyle). External autograft support was performed in all 4 patients in whom a Ross procedure was performed, with an annuloplasty added in 2 patients as part of the Ross procedure. Two patients underwent concomitant procedures, one with septal myectomy and the other with coronary artery bypass graft surgery.

All 5 patients had undergone previous cardiac surgery, and 2 patients had multiple aortic valve and root procedures. Previous surgeries included aortic valve commissurotomy (n = 3), LVOT enlargement and subaortic resection (n = 2), mechanical valve replacement (n = 2), closure of a ventricular defect (n = 1), pulmonary banding (n = 1), correction of an aortic coarctation (n = 1), and correction of an interrupted aortic arch (n = 1). Two patients with a unicuspid aortic valve underwent more than 1 balloon valvuloplasty.

### Early Results

The median myocardial ischemia time was 93 minutes (IQR, 87-95 minutes), and median extracorporeal circulation time was 131 minutes (IQR, 130-138 minutes) (Table 2). There were no early deaths, myocardial infarctions, or neurologic complications. No patient required permanent pacemaker implantation, and there were no early reoperations. Two patients had AR 1 at discharge. One patient had a mean gradient of 15 mm Hg across the aortic valve, which has remained stable over 12 years of follow-up.

**Table 2:** Perioperative data

Patient	Surgery	Concomitant procedure	Annuloplasty	External sinus stabilization	Ascending aortic replacement	Aortic valve reoperation	Sinus rhythm	Death
1	Ross procedure	-	Yes	Yes	-	-	Yes	-
2	Ross procedure	Yes∗	Yes	Yes	-	-	Yes	-
3	Ross procedure	-	-	Yes	-	-	Yes	Yes (auto accident)
4	Ross procedure	Yes∗	-	Yes	-	-	Yes	-
5	Xenograft root replacement	Yes†	-	N/A	-	-	Yes	-

∗ Septal myectomy.

† Coronary artery bypass single graft.

The LVOT diameter increased from preoperative to directly postoperative by a minimum of 6 mm to a maximum of 10 mm, from a median of 12 mm (IQR, 9.5-12.6 mm) preoperatively to a median of 20 mm (IQR, 20-21 mm) at discharge. The median annular diameter was 17 mm (IQR, 15-18 mm) preoperatively and 21 mm (IQR, 20-22 mm) at discharge. The 2 pediatric patients had *z*-scores of −2 and −2.1 preoperatively and −0.99 and 0.04, respectively, at discharge. The median LVOT gradient was reduced from 50.5 mm Hg (IQR, 41.5-57.5 mm Hg) preoperatively to 9 mm Hg (IQR, 7-16 mm Hg) at discharge.

### Late Results

There was 1 late death, a patient who died in an auto accident 3 years postoperatively. Freedom from cardiac death was 100% at 5 years and 10 years. No patients required reoperation on the aortic valve or LVOT. Freedom from aortic valve or LVOT reoperation was 100% at 5 years and 15 years.

One patient underwent reoperation for endocarditis of the pulmonary homograft after 6.5 years. The infected valve was replaced with another pulmonary homograft; the autograft was competent and did not require intervention. The patient has experienced no complications since that operation.

### Echocardiographic Data

At last follow-up, the mean systolic aortic valve gradient was 11 ± 5 mm Hg (range, 3-17 mm Hg) (Table 3). One patient had a mild increase in mean gradient from 10 mm Hg at discharge to 17 mm Hg at 15.4 years postoperatively. The median LVOT diameter remained constant over time, at a median of 20 mm (IQR, 20-21 mm). In addition, aortic annular diameter remained constant, with a median of 25 mm (IQR, 24-26 mm) at last follow-up. In the 2 pediatric patients, *z*-scores at last follow-up were 1.6 and −0.26. Three patients had AR 1 at last follow-up, between 12.1 and 13.4 years postoperatively (Table 3), and 2 of these patients had AR 1 at discharge.

**Table 3:** Postoperative data

Patient	Follow-up, y	Sinus rhythm	PQ and QRS	Last AR	Last mean gradient, mm Hg	Last annulus diameter, mm	Last sinus diameter, mm	Last septal diameter, mm	LVOT at discharge, mm	Last LVOT, mm
1	15.4	Yes	Normal	1	17	25	34	12	20	21
2	12.9	Yes	Normal	1	15	26	36	13	20	20
3	3	Yes	Normal	1	7	26	32	7	20	21
4	5.3	Yes	Normal	0	5	19	26	9	19	19
5	1.2	Yes	Normal	0	9	25	32	11	18	17

*AR*, Aortic regurgitation, *LVOT*, left ventricular outflow tract.

Left ventricular ejection fraction ranged from 62% to 76% at last follow-up. All patients remained in sinus rhythm with normal PR intervals and QRS complexes (Table 3).

## Discussion

Obstruction of the LVOT with hypoplasia of the aortic annulus is a challenging problem. After isolated aortic valve replacement, it can lead to residual pressure load of the left ventricle with its negative consequences,^1^^,^^19^ including increased left ventricular workload and decreased left ventricular mass regression, which have been correlated with increased mortality.^1^^,^^2^ This increased transvalvular gradient may be further increased under stress, particularly in younger patients.^1^^,^^3^

Various techniques to address annular hypoplasia have been developed. Root replacement generally allows for implantation of a larger prosthesis, particularly with stentless prostheses. In addition, use of a pulmonary autograft is associated with low gradients, particularly when implanted as root replacement. This hemodynamic benefit will suffice to achieve an adequate result in borderline annular hypoplasia. In the presence of annular hypoplasia, it has been combined with a limited posterior incision of the annulus,^10^ even though hemodynamic data on that combination are lacking.

Among annular enlargement procedures, the Nicks procedure was the first technique proposed. In this procedure, the aortic incision is made posteriorly and obliquely through the noncoronary aortic sinus and across the aortic annulus into the fibrous origin of the mitral valve.^4^^,^^11^ The incision is closed with a patch, enlarging the annulus and allowing for implantation of a larger prosthesis. In the Manougian procedure,^9^^,^^11^ the aortic incision is extended through the origin of the noncoronary sinus and into the anterior mitral valve leaflet,^5^ with a risk of producing mitral valve regurgitation^5^^,^^6^ In effect, these approaches allow for implantation of a prosthesis 1 or at most 2 sizes larger than the original annular size.

More recently, an aggressive technique of annular enlargement has been proposed.^12^ This involves a Y-incision through the left noncommissure and extending it below the aortic annulus into the area between the nadirs of the left coronary and noncoronary cusps.^12^ This technique enlarges the aortic annulus to a greater degree compared to the Nicks or Manouguian techniques by replacing the entire aortomitral curtain.^12^ An apparent drawback is the potential for distorting the left coronary artery.^12^ Although the formal size of the annulus makes it a seemingly attractive solution, it does not address the LVOT.

The true challenge is the hypoplasia not only of the aortic annulus, but also of the LVOT. Thus far, the Konno technique has been the standard approach to this problem. The LVOT is incised and enlarged through insertion of a patch on its septal side. Because this alters outflow tract anatomy, the incision and enlargement then must be continued into the RVOT to avoid RVOT obstruction. Because most patients needing such an operation are young,^18^ the valve is commonly replaced with a pulmonary autograft as a combined Ross-Konno procedure.^8^^,^^9^^,^^18^ This technique is effective in enlarging both the hypoplastic aortic annulus and the LVOT; however, early mortality ranges between 12.4% and 21.6%.^22^ The septal incision in this procedure carries a risk of injury to the conduction system with atrioventricular block or ventricular arrhythmia, with a 30% rate of pacemaker implantation reported in 2 studies.^10^^,^^17^

Despite the complexity of the procedure, acceptable long-term results have been published,^9^ with some sequelae. Ventricular dyssynchrony has been observed in up to 85%.^23^^,^^24^ Both pacemaker dependency and dyssynchrony may contribute to the development of heart failure and increased late mortality.^23^^,^^24^ Ventricular dyssynchrony is most likely related to interference with septal function; its avoidance ultimately may result in better preservation of long-term function and possibly improve long-term survival. Thus, the procedure is effective but at the cost of relevant morbidity.

It appears desirable to use a technique associated with lesser complexity, cardiac trauma, and minimal interference with the long-term physiology of the left ventricle. Any potential alternative must be sufficiently effective in enlarging the LVOT; in effect, the space between the anterior mitral leaflet and the septum must be increased. Our current approach, an extension of the annular incision used previously,^20^ achieves the goal of sufficient enlargement of both the annulus and the LVOT. We were impressed by the extent to which the space between the anterior mitral leaflet and septum opened up when the posterior annular incision was extended into the left ventricular wall. The postoperative echocardiographic dimensions of the LVOT were normalized in relation to the patient's body surface area, and thus normal hemodynamics were achieved in all instances. Theoretically, an even larger patch could be implanted for even more aggressive enlargement; we did not believe this to be necessary in our cases. The reduction in transvalvular gradients confirms the functional benefits of this technique.

Physiologically, this approach of posterior enlargement bears the lowest risk of impairment of left ventricular function. Even though the cohort was small, normal left ventricular function was observed in all patients. The location of the incision is such that an atrioventricular block should be anatomically impossible. This was confirmed by the postoperative results in our patients. Thus, this new technique appears to be a functionally promising and physiologic alternative to the Ross-Konno procedure.

### Limitations

Owing to the low prevalence of LVOT hypoplasia, the number of patients is very limited. A larger patient cohort with long-term follow-up will be needed to judge the true benefit of our technique. Although we have not observed any specific complications, some may be encountered with more patients thus treated.

## Conflict of Interest Statement

The authors reported no conflicts of interest.

The *Journal* policy requires editors and reviewers to disclose conflicts of interest and to decline handling or reviewing manuscripts for which they may have a conflict of interest. The editors and reviewers of this article have no conflicts of interest.

## Supplementary Data

Video 1 Intraoperative video demonstrating our technique. The video begins at the incision of the aortic annulus and ends with the patch implantation. Transesophageal echocardiography images are included for diameters. Video available at: https://www.jtcvs.org/article/S2666-2507(25)00453-5/fulltext.
