# Supplementary figures and images for: Posterior aortoventricular enlargement of root and outflow tract: Early and mid-term results
Source: JTCVS Tech. 2025 Oct 13;35:102118. doi: 10.1016/j.xjtc.2025.10.001 (PMC12881737; doi:10.1016/j.xjtc.2025.10.001)

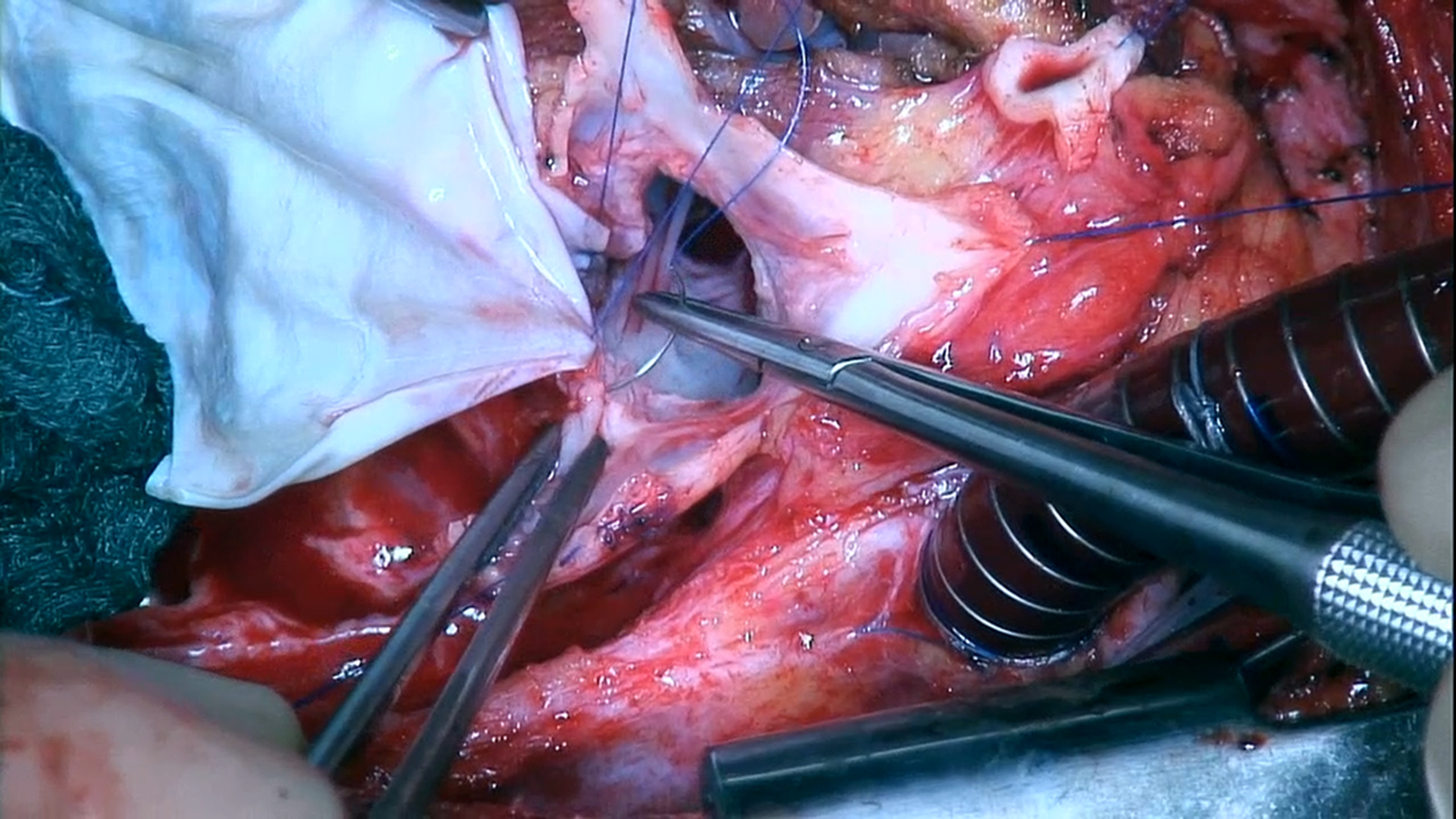

Supplement: Video 1 — Intraoperative video demonstrating our technique. The video begins at the incision of the aortic annulus and ends with the patch implantation. Transesophageal echocardiography images are included for diameters. Video available at: https://www.jtcvs.org/article/S2666-2507(25)00453-5/fulltext. [file fx2.jpg]
